# Genetic determinants controlling maize rubisco activase gene expression and a comparison with rice counterparts

**DOI:** 10.1186/s12870-019-1965-x

**Published:** 2019-08-14

**Authors:** Yu Zhang, Yong Zhou, Qian Sun, Dexiang Deng, Huanhuan Liu, Saihua Chen, Zhitong Yin

**Affiliations:** Jiangsu Key Laboratory of Crop Genetics and Physiology/Co-Innovation Center for Modern Production Technology of Grain Crops/Key Laboratory of Plant Functional Genomics of the Ministry of Education/Jiangsu Key Laboratory of Crop Genomics and Molecular Breeding, Yangzhou University, Yangzhou, 225009 China

**Keywords:** Rubisco activase (RCA), Photosynthesis, Maize, Rice, Quantitative trait locus (eQTL), Promoter

## Abstract

**Background:**

Rubisco activase (RCA) regulates the activity of Rubisco and is a key enzyme of photosynthesis. RCA expression was widely reported to affect plant photosynthesis and crop yield, but the molecular basis of natural variation in RCA expression in a wide range of maize materials has not been fully elucidated.

**Results:**

In this study, correlation analysis in approximately 200 maize inbred lines revealed a significantly positive correlation between the expression of maize RCA gene *ZmRCAβ* and grain yield. A genome-wide association study revealed both *cis*-expression quantitative trait loci (*cis*-eQTLs) and *trans*-eQTLs underlying the expression of *ZmRCAβ*, with the latter playing a more important role. Further allele mining and genetic transformation analysis showed that a 2-bp insertion and a 14-bp insertion in the promoter of *ZmRCAβ* conferred increased gene expression. Because rice is reported to have higher RCA gene expression than does maize, we subsequently compared the genetic factors underlying RCA gene expression between maize and rice. The promoter activity of the rice RCA gene was shown to be stronger than that of the maize RCA gene, suggesting that replacing the maize RCA gene promoter with that of the rice RCA gene would improve the expression of RCA in maize.

**Conclusion:**

Our results revealed two DNA polymorphisms regulating maize RCA gene *ZmRCAβ* expression, and the RCA gene promoter activity of rice was stronger than that of maize. This work increased understanding of the genetic mechanism that underlies RCA gene expression and identify new targets for both genetic engineering and selection for maize yield improvement.

**Electronic supplementary material:**

The online version of this article (10.1186/s12870-019-1965-x) contains supplementary material, which is available to authorized users.

## Background

Ribulose-1,5-bisphosphate carboxylase/oxygenase (Rubisco) catalyzes the first step of photosynthetic carbon assimilation and is a rate-limiting enzyme of photosynthesis. In higher plants, Rubisco activity is regulated by a second enzyme named Rubisco activase (RCA) [1–6]. RCA removes inhibitory sugar phosphates from the active sites of Rubisco in an ATP-dependent way and thus activates Rubisco [7]. The endogenous levels of RCA have been widely reported to affect plant photosynthesis and crop yield. In rice, RCA plays an important role in the regulation of non-steady-state photosynthesis [8]. Overexpression of the RCA gene improves rice grain yield, whereas anti-sense expression shows the opposite effect [9, 10]. In wheat, RCA expression showed a significantly positive linear correlation with plant productivity [11]. In soybean, the expression levels of two RCA genes showed significant correlations with photosynthesis and grain yield [12, 13]. In maize, high-yielding population showed higher RCA expression [14, 15], and the RCA expression displayed significant and positive correlations with grain yield in a wide range of maize inbred lines [16].

Characterization of the genetic elements regulating RCA gene expression could facilitate the modulation of the RCA gene for improving crop yield. For this purpose, genetic determinants that control the expression of RCA genes have been identified in various crops. Functional analysis of the rice RCA gene promoter revealed several *cis*-elements that regulate its expression [17]. Expression quantitative trait locus (eQTL) mapping identified several eQTLs that control the expression of soybean RCA genes [12, 13]. A screen of a leaf cDNA library using a yeast one-hybrid (Y1H) system identified two bZIP transcription factors that interact with the promoter of the soybean RCA gene [18]. Maize is one of the most important C_4_ crops worldwide. Although C_4_ monocot plants including maize show higher photosynthetic efficiency due to an extra biochemical CO_2_-concentrating mechanism (CCM) yielding higher CO_2_ partial pressure (*p*CO_2_) at Rubisco catalytic sites, their Rubisco activation status is generally lower than that of C_3_ monocot plants [19]. Therefore, there should be much room to increase maize Rubisco activation state and subsequently photosynthesis. Because of the primary role of RCA to activate Rubisco in vivo, mapping the genetic factors underlying RCA expression in maize is of paramount importance.

Recently, based on linkage analysis using a maize recombinant inbred line (RIL) population derived from two parental inbred lines, we identified several eQTLs underlying RCA gene expression [20]. Although these eQTLs can help to understand the regulation of the RCA gene in maize, the molecular basis of natural variation in RCA expression in a wide range of maize materials has not been fully elucidated. Genome-wide association analysis (GWAS) based on linkage disequilibrium (LD) studies in a natural population allows for evaluation of multiple alleles underlying the natural variation of a target trait. Additionally, GWAS can increase resolution and facilitate gene discovery [21].

Rice, one of the most important C_3_ crops, shows a higher level of RCA gene expression than that of maize [22]. The difference in RCA gene expression between maize and rice most likely required the evolution of a different regulatory mechanism. Therefore, the higher level of RCA gene expression in rice than that in maize suggests that rice might have stronger RCA gene regulatory factors, in the form of either *cis*- or *trans*-regulatory factors, than maize. To date, no information is available on the similarities or differences in the RCA gene regulatory elements between maize and rice. If rice is confirmed to have a stronger RCA regulator than maize, future high-yield breeding of maize can focus on replacing its weaker RCA gene regulator with the stronger one from rice.

In maize, two RCA genes, *ZmRCAα* and *ZmRCAβ*, have been identified and cloned. The *ZmRCAβ* expression level was approximately 10-fold that of *ZmRCAα*. Additionally, compared with *ZmRCAα*, *ZmRCAβ* showed a much higher correlation with grain yield [16]. In most studies, only one RCA gene, *OsRCA*, has been identified in the rice genome [23]. Alternative splicing of this gene produces two RCA isoforms, the short β form and the long α form [24]. In this study, GWAS was employed to map eQTLs for the maize RCA gene, *ZmRCAβ*, and the rice RCA gene, *OsRCA*. Subsequently, sequencing of target DNA segments and candidate gene association were employed to screen for elite alleles of the RCA gene promoter in these two species. Finally, genetic transformation experiments were conducted to examine the differences in RCA gene promoter activity between maize and rice. Our findings showed that a 2-bp insertion and a 14-bp insertion in the promoter of *ZmRCAβ* conferred increased gene expression and that the rice RCA gene promoter had higher activity than that of the maize RCA gene promoter.

## Results

### Variation of *ZmRCAβ* expression and its correlation with grain yield

**Fig. 1:**
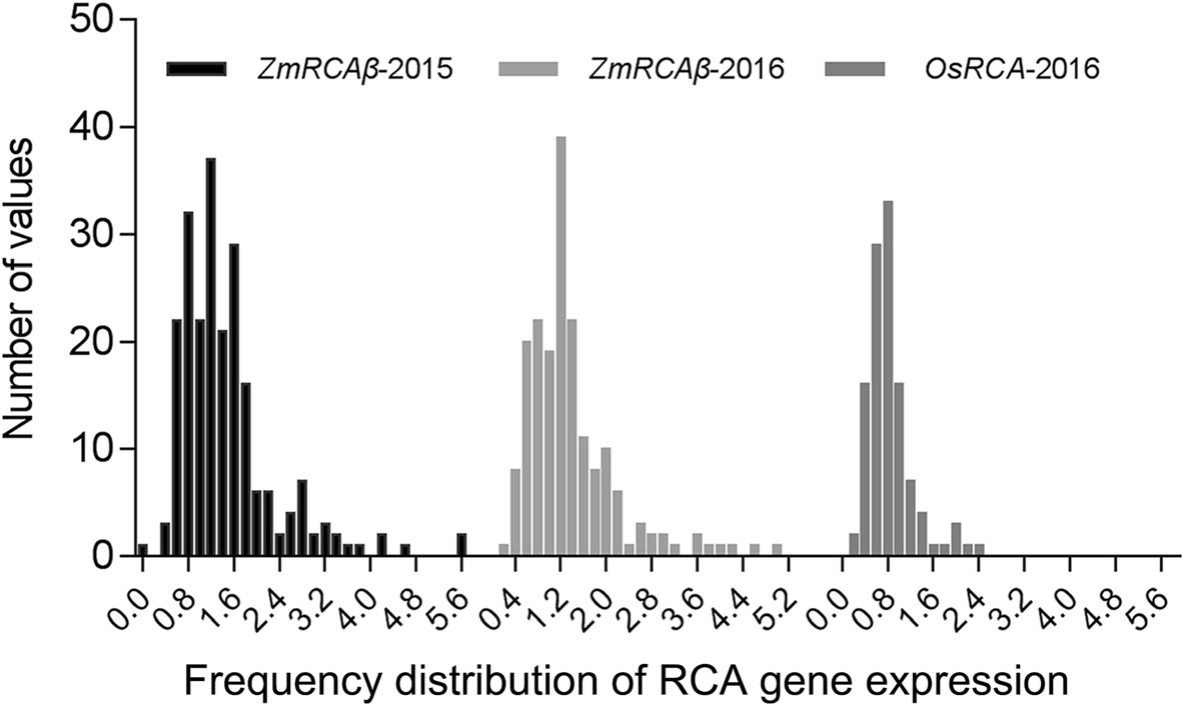
Frequency distribution of the expression of *ZmRCAβ* and *OsRCA*

Because alternative splicing of the *ZmRCAβ* precursor RNA creates two transcripts [16], we measured the transcript expression of this gene by designing primers that detect the two transcripts simultaneously (Additional file 1: Table S1). The transcript expression of *ZmRCAβ* and grain yield exhibited wide phenotypic variations in the maize association populations (Table 1 and Fig. 1). The means, standard deviations, ranges, skewness, broad-sense heritability values, and analysis of variance (ANOVA) for *ZmRCAβ* transcript expression and grain yield are presented in Table 1. The average relative value of *ZmRCAβ* transcript expression were 1.46 and 1.38 in 2015 and 2016 growing season, respectively. The variation range of *ZmRCAβ* expression was larger, 95% of lines fell in the range of 0.21–3.13 in 2015 and 0.17–2.93 in 2016. The average of grain yield were 45.60 and 45.11 g per plant (g/plant) in 2015 and 2016 growing season, respectively. The variation range of grain yield was larger, 95% of lines fell in the range of 19.83–71.37 g/plant in 2015 and 17.32–72.90 g/plant in 2016. ANOVA indicated that *ZmRCAβ* transcript expression and grain yield were significantly affected by genotype (*P* < 0.01). The heritability (*H*^*2*^) estimates for *ZmRCAβ* transcript expression ranged from 75 to 80%, and for grain yield from 76 to 86% (Table 1). Overall, the transcript expression of *ZmRCAβ* and grain yield clearly exhibited wide natural variation in the maize association populations.

**Table 1:** Descriptive statistics, ANOVA and broad-sense heritability for RCA gene expression and grain yield

Trait	Mean ± SD	Range	Skew	G^a^	R^b^	*H*^*2*^ (%)^c^
*ZmRCAβ*-2015	1.46 ± 0.85	0.09–5.66	2.01	s	ns	0.75
*ZmRCAβ*-2016	1.38 ± 0.79	0.10–4.92	1.90	s	ns	0.80
ZmYield-2015	45.60 ± 13.15	11.00–83.95	0.10	s	ns	0.86
ZmYield-2016	45.11 ± 14.18	13.38–93.78	0.53	s	ns	0.76
*OsRCA*-2016	0.94 ± 0.54	0.20–2.55	1.15	s	ns	0.64

Note: The *ZmRCAβ* and *OsRCA* expression data are represented by the relative values compared to their reference genes, *ZmActin* and *OsActin*, respectively. The unit for grain yield is gram per plant (g/plant). *ZmRCAβ* expression measured in 2015 and 2016 is termed as *ZmRCAβ*-2015 and *ZmRCAβ*-2016, respectively. Maize grain yield measured in 2015 and 2016 is termed as ZmYield-2015 and ZmYield-2016, respectively. *OsRCA* expression measured in 2016 is termed as *OsRCA*-2016

s: significant difference at *P* < 0.01; ns: not significant

^a^ Genotype;

^b^ Replication;

^c^ Broad-sense heritability

**Fig. 2:**
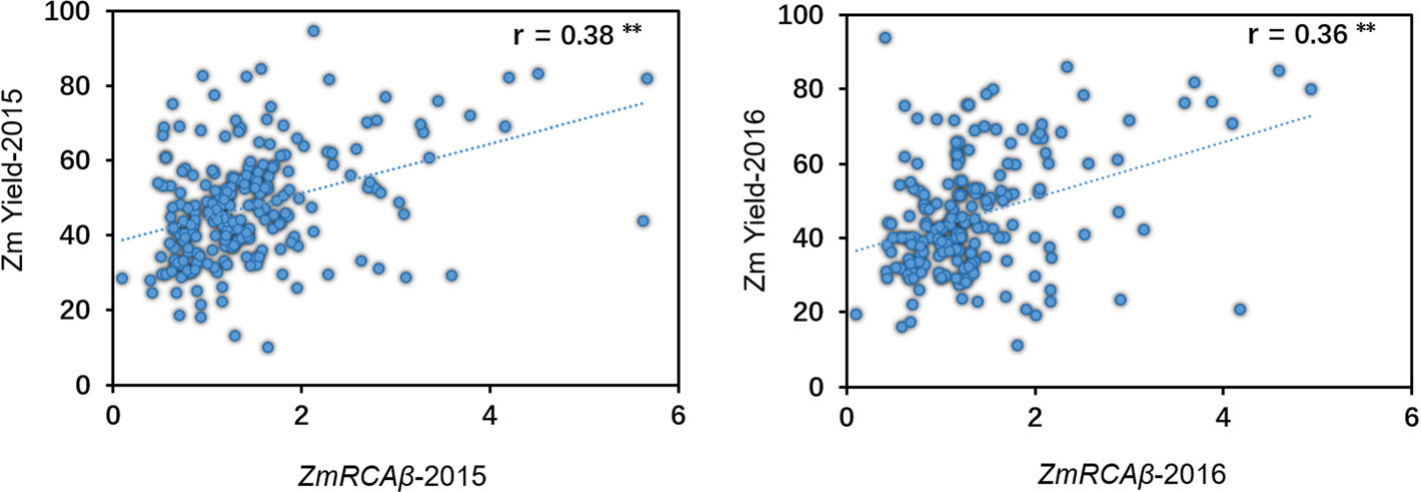
Correlation analysis for the transcript expression of *ZmRCAβ* and the grain yield in the association population. ∗∗ significant at *P* < 0.01

In our previous study, we determined the transcript and protein expressions of *ZmRCAβ* and a newly discovered RCA gene *ZmRCAα* in 123 maize inbred lines. The transcript expression of *ZmRCAβ* was observed to correlate well with its protein expression; both the transcript and protein expressions of *ZmRCAβ* were significantly correlated with grain yield, and more importantly, the correlations were much higher than those between *ZmRCAα* and grain yield [16]. In the current study, we focused on *ZmRCAβ* transcript expression, and observed that during the two growing seasons of 2015 and 2016, the *ZmRCAβ* transcript expression also displayed a significant correlation with the corresponding grain yield (*P* < 0.01). Pearson phenotypic correlation coefficient between *ZmRCAβ* transcript expression and grain yield was 0.38 in the 2015 growing season and 0.36 in the 2016 growing season (Fig. 2). The above results indicate that the *ZmRCAβ* transcript expression could affect maize grain yield.

### Genome-wide association study for *ZmRCAβ* expression

**Fig. 3:**
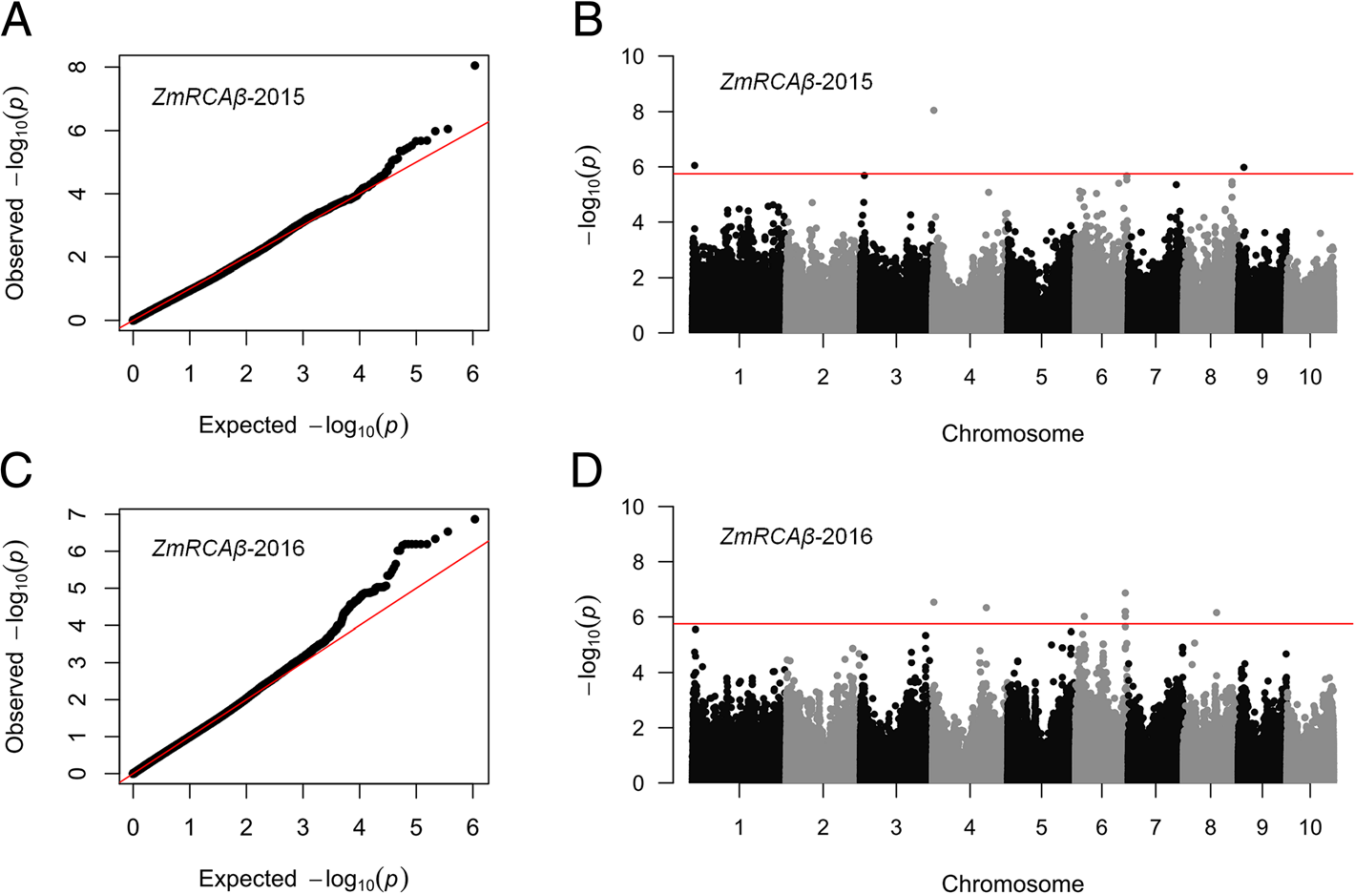
GWAS for the expression of *ZmRCAβ*. **a**, **c** Quantile-quantile plot for the GWAS under a mixed linear model (MLM). **b**, **d** Manhattan plot for the GWAS. Note: The red line indicates a significant association signal (−log*P* > 5.75)

To identify the genetic loci associated with *ZmRCAβ* expression, GWAS was performed by using 558,629 genomic single nucleotide polymorphisms (SNP) and the *ZmRCAβ* transcript expression data generated in an association population comprising 222 and 182 maize lines in 2015 and 2016, respectively (see Plant materials and methods). To avoid spurious associations, such as the historical relationships and selection patterns of the maize lines in the association population, multiple statistical models were evaluated in the GWAS. Visual observation of quantile-quantile (Q-Q) plots provides an indication of the accuracy of the model used to analyze the data. As shown in the Q-Q plots (Fig. 3A, C), the *P*-values of the mixed linear model (MLM) were close to the expected values, indicating this model was suitable for reducing the effects of population structure on *ZmRCAβ* transcript expression. Thus, we conducted GWAS for *ZmRCAβ* transcript expression with the MLM model.

Three and 12 SNPs were identified with significant marker-trait associations with *ZmRCAβ* transcript expression at the Bonferroni-adjusted significance threshold in 2015 and 2016 growing seasons, respectively (Table 2 and Fig. 3B, D). These SNPs were distributed on chromosomes 1, 4, 6, 8, and 9, and the phenotypic variation explained by each SNP ranged from 13.65 to 29.71%. Interestingly, GWAS for grain yield showed that the significant marker chr6.S-28129864 for *ZmRCAβ* expression (Table 2) was also significantly associated with grain yield (Additional file 9: Table S6). Of the significant marker-trait associations for *ZmRCAβ* expression, the marker PZE-104005992 was repeatedly detected in 2015 and 2016 growing seasons. A survey of the maize reference genome sequence (https://www.maizegdb.org/) revealed that this marker was located at 3.6 Mb upstream of *ZmRCAβ* on chromosome 4.

**Table 2:** SNP markers associated with the expression of *ZmRCAβ* in maize association population

Trait	Marker	Chromosome	Position	–log*P*	*R* ^*2*^ *(%)*
*ZmRCAβ-*2015	PZE-104005992	4	4,289,239	8.05	24.87
	chr1.S_7,076,785	1	7,076,785	6.05	13.98
	chr9.S_18,890,334	9	18,890,334	5.98	13.65
*ZmRCAβ*-2016	chr6.S_159,773,778	6	159,773,778	6.87	20.31
	PZE-104005992	4	4,289,239	6.53	29.71
	chr4.S_172,763,634	4	172,763,634	6.34	18.74
	chr6.S_159,772,882	6	159,772,882	6.19	17.93
	chr6.S_159,772,884	6	159,772,884	6.19	17.93
	chr6.S_159,772,951	6	159,772,951	6.19	17.93
	chr6.S_159,773,131	6	159,773,131	6.19	17.93
	chr6.S_159,773,245	6	159,773,245	6.19	17.93
	chr6.S_159,773,246	6	159,773,246	6.19	17.93
	chr8.S_107,014,893	8	107,014,893	6.16	17.65
	chr6.S_28,129,864	6	28,129,864	6.02	17.26
	chr6.S_159,772,707	6	159,772,707	6.02	17.75

Significant association: -log*P* > 5.75

### Candidate gene association analysis of the polymorphisms in the promoter region of *ZmRCAβ*

The 1700-bp region of the *ZmRCAβ* promoter, which was previously shown to be sufficient for the proper regulation of expression in plants [25, 26], was cloned and sequenced in the maize association population. The promoter sequences of 186 inbred lines of different geographic origins were successfully cloned and sequenced. Multiple sequence alignment of the promoter region revealed 79 polymorphic sites in the 186 maize inbred lines (Additional file 2: Table S2). To determine whether these sequence polymorphisms were related to the observed variation in *ZmRCAβ* transcript expression, candidate-gene association analysis was performed. Two polymorphisms, a 2-bp indel and 14-bp indel, were repeatedly detected to be significantly associated with the expression of *ZmRCAβ* in both 2015 and 2016 growing seasons at *P* < 0.01 (Table 3). The 2-bp indel located 144-bp upstream of the start codon of *ZmRCAβ* explained 4.24 and 6.15% of the phenotypic variation in 2015 and 2016, respectively. The 14-bp indel located 210-bp upstream of the start codon of *ZmRCAβ* explained 3.80 and 5.98% of the phenotypic variation in 2015 and 2016, respectively.

**Table 3:** Polymorphisms in the *ZmRCAβ* promoter region significantly associated with *ZmRCAβ* expression in maize association population

Polymorphic type	Position	–log*P*		*R* ^*2*^ *(%)*	
		2015	2016	2015	2016
2-bp indel	−114	2.32	3.13	4.24	6.15
14-bp indel	−210	2.12	3.05	3.80	5.98

Significant association: -log*P* > 2

### Haplotype analysis for *ZmRCAβ* promoter

**Fig. 4:**
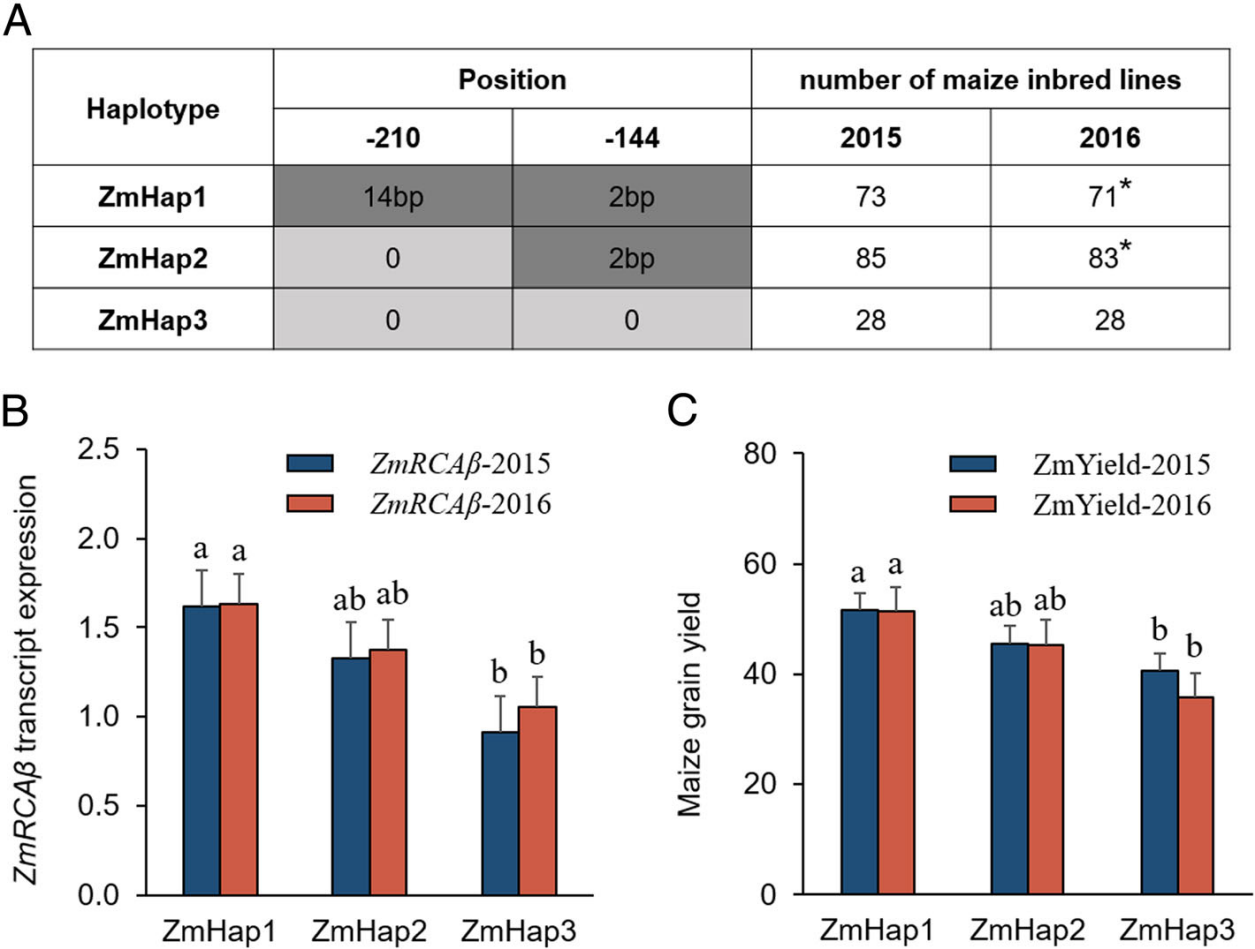
*ZmRCAβ* promoter haplotypes and the average *ZmRCAβ* expression and grain yield for each haplotype. **a** Haplotype analysis of the *ZmRCAβ* promoter regions. The dark gray-shaded cells represent the favorable alleles. *Two lines harboring ZmHap1 and two lines harboring ZmHap2 used for determining *ZmRCAβ* expression and grain yield were not available in 2016. **b**, **c** The average expression of *ZmRCAβ* and maize grain yield for different haplotypes. Error bars represent the standard error. The number of data points used for calculating the standard error is the product of number of maize lines belonging to each haplotype and biological replicates of each line. Multiple comparison of phenotypic data was performed using Fisher’s protected Least Significant Difference (LSD) test. Different English alphabet means significant difference at *P* < 0.05

Based on the polymorphisms significantly related with RCA expression, the promoter sequence of *ZmRCAβ* was divided into 3 haplotypes, ZmHap1, ZmHap2 and ZmHap3 (Fig. 4A). The inbred lines harboring each of these haplotypes are shown in Additional file 3: Table S3. The inbred lines carrying the ZmHap1 showed the highest *ZmRCAβ* expression and the highest grain yield, and those carrying the ZmHap3 showed the lowest *ZmRCAβ* expression and the lowest grain yield (Fig. 4B, C).

**Fig. 5:**
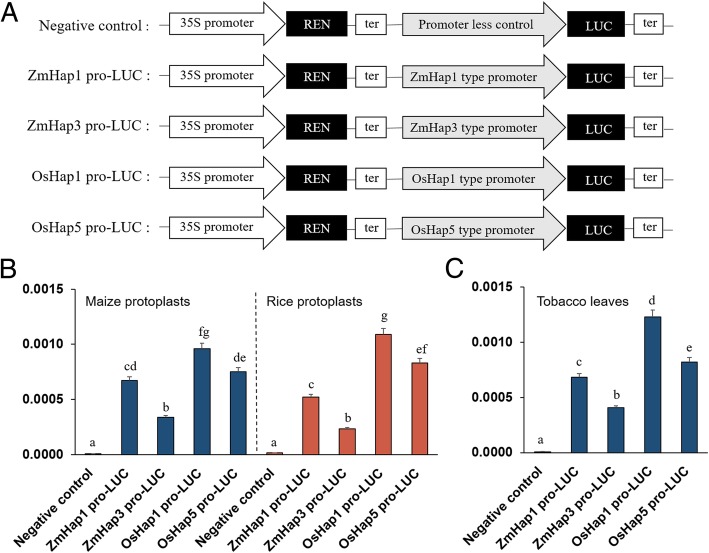
Activity of different types of RCA promoters. **a** Schematic diagrams of the dual luciferase assay vector. REN, Renilla luciferase; LUC, firefly luciferase; ter, terminator. **b** Ratios of LUC and REN activity in maize and rice protoplasts transformed with recombinant plasmids containing different types of *ZmRCAβ* and *OsRCA* promoters and negative control. **c** Ratios of LUC and REN activity in tobacco (*Nicotiana benthamiana*) leaves transformed with recombinant plasmids containing different types of *ZmRCAβ* and *OsRCA* promoters and negative control. Multiple comparison of phenotypic data was performed using LSD test. Different English alphabet means significant difference at *P* < 0.05

A dual-luciferase reporter system was used to verify the ZmHap1 promoter of *ZmRCAβ* had higher activity than that of the ZmHap3 promoter. We cloned ZmHap1 and ZmHap3 promoters from inbred lines CIMBL57 and CIMBL88, respectively. These two inbred lines showed the highest and lowest levels of *ZmRCAβ* expression, respectively. The ZmHap1 or ZmHap3 promoter-driven LUC (ZmHap1 or ZmHap3 pro-LUC) and CaMV35S promoter-driven REN (35S-REN; as an internal control) were constructed in the same plasmid (Fig. 5A), and expressed in maize protoplasts, rice protoplasts, and tobacco *(Nicotiana benthamiana)* leaves. The dual luciferase expression system with no promoter to drive LUC was used as a negative control (Fig. 5A). The LUC/REN ratio, which reflects in vivo promoter activity, was monitored. The LUC/REN value of ZmHap1 promoter was significantly higher than that of the ZmHap3 promoter in maize protoplasts, rice protoplasts, and the leaves of tobacco (Fig. 5B, C). We also compared the activity of ZmHap1 and ZmHap3 promoters using rice callus genetic transformation experiment, and observed a similar result (Additional file 4: Figure S1). These results confirmed that the ZmHap1 promoter had higher activity than that of the ZmHap3 promoter. Therefore, ZmHap1 can be regarded as an elite allele for the *ZmRCAβ* promoter.

### Comparison of RCA gene regulatory elements between maize and rice

To compare the regulating strength of RCA gene expression determinants between maize and rice, the transcript expression of rice RCA gene *OsRCA* was determined in an association population containing 114 cultivars with extensive genetic diversity, and the genetic determinants underlying the transcript expression of *OsRCA* were identified using the same method employed in maize as described above. Similar to the measurement of *ZmRCAβ* expression, because alternative splicing of the *OsRCA* precursor RNA produces two transcripts [24], we also measured the transcript expression of this gene by designing primers that detect the two transcripts simultaneously (Additional file 1: Table S1). The transcript expression of *OsRCA* exhibited wide natural variations in the rice association population (Table 1 and Fig. 1). A 12.75-fold difference occurred among the cultivars in the rice association population (Table 1). GWAS using the rice association population revealed 5 SNPs significantly associated with *OsRCA* transcript expression (Additional file 5: Table S4 and Additional file 6: Figure S2).

The promoter sequence of 1500-bp was cloned and sequenced in 107 rice cultivars. Candidate-gene association analysis revealed 12 polymorphisms significantly associated with *OsRCA* transcript expression in its promoter region (Additional file 7: Table S5). Based on these polymorphisms, the *OsRCA* promoter was divided into 5 haplotypes (Additional file 3: Table S3). The cultivars carrying OsHap1 had the highest *OsRCA* expression and those carrying OsHap5 had the lowest *OsRCA* expression (Additional file 8: Figure S3B).

The aforementioned experiments of protoplast transient expression, leaf transient expression, and callus genetic transformation were conducted to compare the promoter activity of maize *ZmRCAβ* and rice *OsRCA*. We cloned OsHap1 and OsHap5 promoters from rice cultivars Jing 185–7 and Saber, respectively. These two cultivars showed the highest and lowest levels of *OsRCA* expression, respectively, in the rice association population. Each of the OsHap1 and OsHap5 promoter sequences was fused with the LUC or GUS reporter gene in plasmid (Fig. 5A and Additional file 4: Figure S1A). In the protoplast and leaf transient expression experiments, the LUC/REN value of OsHap1 was significantly higher than that of OsHap5, confirming that the OsHap1 promoter had higher activity than that of the OsHap5 promoter (Fig. 5B, C). The LUC/REN value of rice stronger OsHap1 promoter was significantly higher than maize stronger ZmHap1 promoter. Notably, the LUC/REN value of the rice weaker OsHap5 promoter was even higher than the maize stronger ZmHap1 promoter although the difference observed in maize protoplast experiment was not significant (Fig. 5B, C). The similar results were also observed in the callus genetic transformation experiment (Additional file 4: Figure S1B). These results suggested that the activity of the rice RCA promoter was higher than that of the maize RCA promoter.

## Discussion

### Mapping of favorable alleles underlying *ZmRCAβ* expression for maize high-yield breeding

Strong evidence exists that two traits are causally related when the two traits are significantly correlated with one another and have coincident QTLs that are consistent with the correlation [27]. In the present study, the expression level of *ZmRCAβ* was positively correlated with grain yield in an association population consisting of approximately 200 maize inbred lines. Furthermore, GWAS for grain yield showed that one QTL for grain yield coincided with the eQTL for *ZmRCAβ* expression (Additional file 9: Table S6). We also observed similar results in our previous study using 123 maize inbred lines [16]. These data suggest that the RCA gene might be a potential factor affecting plant productivity. Therefore, identifying the key genomic regions that regulate the expression level of *ZmRCAβ* could aid in maize high-yield breeding via the marker-assisted-selection (MAS) method.

The success of MAS breeding depends on the gene mapping resolution and germplasm diversity. In our previous study, we detected five eQTLs for *ZmRCAβ* in a RIL population containing 242 maize lines [20]. However, these eQTLs were mapped to relatively large mapping intervals, and no causal explicit genetic elements could be inferred for regulating *ZmRCAβ* expression. Additionally, these eQTLs do not represent the elite alleles underlying natural variation of *ZmRCAβ* expression because they were mapped in a bi-parental population. GWAS combined with target region association analysis in a diverse association population can provide the opportunity to discover elite alleles of interest and delimit them into a relatively small genomic region [28]. This method takes advantage of current and historical recombination events for gene fine mapping, particularly in maize, a species that shows high diversity and rapid LD decay. Therefore, in the present study, GWAS combined with target region association analysis was employed to discover favorable alleles underlying *ZmRCAβ* expression in a maize association population. The results show that the favorable alleles underlying *ZmRCAβ* expression were finely mapped.

In the present study, GWAS initially detected one major eQTL, PZE-104005992, on chromosome 4 in approximately 200 inbred lines with extensive genetic diversity. This eQTL was repeatedly detected across two field-growth conditions and explained a large phenotypic variation (Table 2). We also detected this eQTL in our previous study [20]. Therefore, PZE-104005992 can be considered as a primary breeding target for modulation of *ZmRCAβ* expression. Subsequently, we focused on refined mapping of this eQTL. Considering that the expression of one gene is usually affected by the sequence variation in its promoter and that the genomic location of the *ZmRCAβ* promoter coincided with the eQTL PZE-104005992, we employed candidate-gene association analysis to the *ZmRCAβ* promoter. The results show that two polymorphic sites, a 2-bp indel and a 14-bp indel, in the promoter were significantly associated with gene expression (Table 3). The inbred lines carrying the 2-bp and 14-bp insertion showed higher *ZmRCAβ* expression and higher yields than those of other inbred lines (Fig. 4). Finally, genetic transformation experiments showed the promoter with the 2-bp and 14-bp insertion had higher activity than that of other promoters (Fig. 5 and Additional file 4: Figure S1). Based on these results, we conclude that the promoter carrying the 14-bp and 2-bp insertion might be a favorable allele for maize high-yield breeding. Functional markers are derived from polymorphic sites within genes that are causally involved in phenotypic trait variation [29]. The 2-bp and 14-bp indels identified in the present study can be used for development of functional markers.

### Genetic mechanism underlying RCA gene expression

When an eQTL is within 5 Mb of its target gene, a *cis*-eQTL is defined; otherwise, the eQTL is a *trans*-eQTL [30–33]. The identification of a *cis*-eQTL suggests that the genetic determinants for gene expression are adjacent to or coincident with the genomic region of the target gene. A *trans*-eQTL indicates that genetic determinants far from the target gene control its expression [34]. In the present study, GWAS revealed 14 and 5 SNPs that were significantly associated with the expression of *ZmRCAβ* and *OsRCA*, respectively (Table 2 and Additional file 5: Table S4). Of the 14 significant SNPs for *ZmRCAβ*, the marker PZE-104005992 was 3.6 Mb from *ZmRCAβ* in the maize reference genome (https://www.maizegdb.org/) and was called a *cis*-eQTL, whereas the other SNPs were *trans*-eQTLs. Of the 5 significant SNPs for *OsRCA*, the marker id11011480 was 0.7 Mb from *OsRCA* in the rice reference genome (http://www.ricedata.cn/gene/) and was a *cis*-eQTL, whereas the other SNPs were *trans*-eQTL. Notably, the *cis*-eQTL showed a much stronger association with gene expression and explained a much larger proportion of phenotypic variation than any individual trans-eQTL in both rice and maize (Table 2 and Additional file 5: Table S4). We also obtained similar results in our previous study: in maize, we identified 1 *cis*-eQTL and 4 *trans*-eQTLs for the expression of *ZmRCAβ* in a RIL population under two field-growth environments and observed that the *cis*-eQTL explained the most phenotypic variation and was stably expressed across the different environments [20]. In soybean, we identified 13 SNPs significantly associated with the expression of the RCA gene *GmRCAβ* and found that the *cis*-eQTL had a stronger association with gene expression than that of the *trans*-eQTLs [12]. These results indicate that the expression of the RCA gene might be controlled by a combination of both *cis*-acting and *trans*-acting eQTLs and that the *cis*-eQTL played a dominant role in regulating RCA gene expression.

Promoters can affect the action of both *cis*- and *trans*-eQTLs on the expression of a target gene via alterations in its *cis*-acting regulatory elements or transcription factor binding sites [34]. Therefore, DNA sequence variation in promoters is a potential factor explaining the diversity of *ZmRCAβ* and *OsRCA* expression. In the present study, candidate-gene association analysis revealed 2 polymorphic sites in the *ZmRCAβ* promoter and 12 polymorphic sites in the *OsRCA* promoter that were significantly associated with gene expression (Table 3 and Additional file 7: Table S5). The RCA gene expression was significantly different in haplotypes of maize inbred lines or rice cultivars divided according to the detected significant association polymorphic sites (Fig. 4 and Additional file 8: Figure S3). Furthermore, genetic transformation experiments confirmed that the two types of promoters of *ZmRCAβ* and *OsRCA* had different activity (Fig. 5 and Additional file 4: Figure S1). These results suggest that promoter sequence variation can influence RCA gene expression. A similar result was also observed in soybean in which sequence variation in the *GmRCAβ* promoter contributed to diversity in *GmRCAβ* expression [12].

### Comparison of the promoter activity between maize and rice provides a potential approach for maize high-photosynthetic-efficiency breeding

In the present study, we used qRT-PCR to determine maize and rice RCA gene expression. Although gene expression can be measured quickly and accurately, this method provides only the relative expression data of the targeted gene compared with a reference gene. Because the absolute values of reference gene expression are not available in maize and rice, we could not compare the RCA gene expression magnitude between these two species. Therefore, we focused on comparing the promoter activity of maize *ZmRCAβ* and rice *OsRCA* using genetic transformation experiments.

Different results have been reported on the cell type-specific expression pattern of maize RCA [35–37]. A high density of immune-gold particles for RCA was observed to be located in chloroplast stroma of both bundle sheath (BS) and mesophyll (M) cells in maize leaf [35]. Similarly, although the authors did not point out, an immunoblot analysis of BS and M cells total proteins in maize revealed that RCA accumulated to a similar level in the two types of cells (according to Fig. 7B in the paper by Wostrikoff et al. [36]). In contrast, maize RCA was shown to express at a much higher level in BS cells than in M cells as revealed by large-scale quantitative proteomics [37] and RNA-seq analysis [38]. If the promoter of *ZmRCAβ* and *OsRCA* has cell type-specific expression pattern between BS and M cells, it should show a different expression magnitude between maize and rice leaf tissues that contain a different ratio of M and BS cell number. In the present study, neither the promoter activity of a maize haplotype, either ZmHap1 or ZmHap3, nor the promoter activity of a rice haplotype, either OsHap1 or OsHap5, showed significantly different activity between maize and rice protoplasts (Fig. 5B). These results suggest that the *ZmRCAβ* and *OsRCA* promoter did not yield significant cell type-specific activity in BS and M cells.

Genetic transformation experiments revealed that the activity of the rice *OsRCA* promoter was significantly higher than that of the maize *ZmRCAβ* promoter (Fig. 5 and Additional file 4: Figure S1). This result is consistent with the finding that the RCA gene in maize showed a lower expression level than that in rice [22]. Compared with maize, rice Rubisco operates in a much lower CO_2_ environment and at a higher activation state [19]. To maintain the high activation state of Rubisco, the rice RCA gene promoter likely evolved a high regulating strength to keep the RCA gene expression at a high level. Considering C_4_ plants have an extra CCM and consequently much higher level of CO_2_ concentration at their Rubisco catalytic sites than C_3_ plants, we attempted to search for the difference in the RCA gene promoter between C_3_ and C_4_ species. A survey of the PlantCARE database [39] showed that, compared with C_4_ species including maize, the promoter from C_3_ species including rice have a significantly higher number of *cis*-acting elements associated with light regulation (Additional file 10: Table S7). Because the RCA gene is highly regulated by light [40], the increase in number of light regulation-related *cis*-elements might lead to an RCA gene promoter with increased activity. Future studies are required to compare the regulating strength of the RCA promoter between C_3_ and C_4_ plants using multiple C_3_ and C_4_ species.

As it was shown that the activity of the rice *OsRCA* promoter was higher than that of the maize *ZmRCAβ* promoter (Fig. 5 and Additional file 4: Figure S1), we suppose that maize photosynthesis could be improved by replacing the maize *ZmRCAβ* promoter with the rice *OsRCA* promoter. This replacement can combine the high CO_2_ environment at the Rubisco catalytic sites with a high RCA gene expression in maize plants. A concern for this replacement is that it might affect the action of some heat-stress related regulatory elements that control maize RCA gene expression. RCA is known to be particularly susceptible to heat stress [41, 42], and it was reported that heat stress induced a new RCA isoform with higher molecular weight in maize [43]. Another concern is that the replacement might lead to a decrease of maize Rubisco content. Recently, it was reported that RCA expression negatively affected Rubisco content in transgenic rice [6]. Further studies are required to investigate the effect of the replacement on maize RCA gene expression, Rubisco content, photosynthesis, and grain yield under both common and heat-stressed conditions.

## Conclusion

This study of eQTL mapping and genetic transformations of promoter regions were performed to characterize the genetic factors underlying the expression of the *ZmRCAβ* gene in maize. Furthermore, we compared the regulating strength of the identified factor between the C_4_-plant maize and the C_3_-plant rice. Our study showed that the RCA gene might play a role in maize grain yield. A 2-bp insertion and a 14-bp insertion in the promoter conferred higher expression of maize RCA gene. The promoter activity of the rice RCA gene was stronger than that of the maize RCA gene. The two identified indels associated with maize RCA gene can be used in marker-assisted selection breeding for high photosynthetic efficiency. More importantly, people usually transfer C_4_-specific genes from C_4_-crop maize to C_3_-crop rice in traditional high-photosynthetic-efficiency breeding. Our study suggested a possible and novel method in high-photosynthetic-efficiency breeding, which is, in the other way around, transferring gene from C_3_-crop rice to C_4_-crop maize.

## Methods

### Plant materials and plant growth conditions

A panel of 437 maize lines described previously [44] was used. All 437 lines were genotyped for SNP markers using high-throughput genotyping platforms, and approximately 558,629 polymorphisms with minor allele frequencies (MAF) ≥0.05 were available for a GWAS [44]. The 437 lines were planted in two growing seasons in 2015 and 2016 on the experimental farm of the Agricultural College of Yangzhou University. In each growing season, the 437 lines were planted in a randomized block design with two replicates. Each line was planted in one row per block. The length of each row was 2.5 m, the spacing between plants in each row was 0.25 m, and the spacing between rows was 0.55 m. Leaf tissues for gene expression measurement were prepared according to our previous study [16]. At 32 d after anthesis, the leaves located nearest to the ear were harvested from three randomly selected plants of each line per block in the morning (9:00–11:30 a.m.) on a sunny day, frozen immediately in liquid nitrogen, and stored at − 80 °C before measuring *ZmRCAβ* expression. To minimize the impacts of differences in plant growth stage on gene expression measurements, we chose 222 and 182 lines with similar anthesis time in 2015 and 2016, respectively. The origin of the selected lines is shown in Additional file 3: Table S3.

A panel of 218 rice cultivars was used to evaluate *OsRCA* gene expression. All 218 cultivars were genotyped for SNP markers using high-throughput genotyping platforms, and approximately 36,900 polymorphisms with MAF ≥ 0.05 were available for GWAS [45]. All cultivars were planted on the experimental farm of the Agricultural College of Yangzhou University in 2016. The cultivars were planted in a randomized block design with two replicates. Each cultivar was planted in one row per block. Row length was 1.5 m, row distance was 0.2 m, and plant distance in each row was 0.132 m. At the late filling stage of development, the rice flag leaves were collected individually from three randomly selected plants of each cultivar per block in the morning (9:00–11:30 a.m.) on a sunny day, frozen immediately in liquid nitrogen, and stored at − 80 °C until further use. To minimize the impacts of differences in growth stage on the measurements of gene expression, we chose 114 cultivars with similar anthesis time. The origin of the selected cultivars is shown in Additional file 3: Table S3.

### Trait measurement and phenotypic data collection

Total RNA was extracted using TRIzol reagent (Vazyme, China). We used HiScript reverse transcriptase (Vazyme, China) to prepare cDNA. Gene expression levels were determined by quantitative real-time PCR (qRT-PCR) using an SYBR Premix Ex Taq kit (Vazyme, China) on a Bio-Rad CFX96 system (Applied Biosystems). The expression of endogenous reference genes *ZmActin* (GenBank accession number J01238) and *OsActin* (GenBank accession number AB047313) was used to normalize the transcript levels in each maize and rice sample, respectively. Gene specific primers (Additional file 1: Table S1) were used for qRT-PCR. The following formula was used to obtain the normalized expression of target gene for each sample: ΔΔC_T_ = (C_T, Target_ – C_T, Actin_) genotype – (C_T, Target_ – C_T, Actin_) calibrator. Each sample was prepared by mixing equal amounts of the collected leaves from the three plants described above for each line (cultivar) per block. Therefore, in each growing season, each maize line or rice cultivar contained two samples from two blocks.

Maize grain yield for each line per block was estimated using the average yield of five plants in the middle of each row. At maturity, the ears of the corresponding plants were hand harvested, dried to a constant mass, and threshed; and the mean grain yield per plant was recorded.

### Statistical analyses of phenotypes

Descriptive statistical analysis and correlation analyses were performed using the statistical software package SPSS Statistics 17.0 for Windows (SPSS, Inc., Chicago, IL, USA). Two-tailed ANOVA was used for comparisons between blocks and genotypes. The broad-sense heritability (*H*^*2*^) for RCA gene expression or grain yield in each growing season was implemented by SAS 9.2 (SAS institute, Cray, USA) and was estimated by the following equation *H*^*2*^ = V_g_/(V_g_ + V_e_/r), where V_g_ and V_e_ represent genetic variance and error variance, respectively, and were calculated from the ANOVA as described above; r is the block replicate number in field experiment. Frequency distribution analysis was performed using GraphPad Prism (version 7, GraphPad Software, San Diego, CA).

### Genome-wide association study

TASSEL software 5.0 was used to perform GWAS [46]. To account for the effects of population structure on the mapping panel and genetic relatedness among panel members, the kinship matrix (K) of the association panel was calculated as previously described [47]. According to the Q-Q plots from the TASSEL 5.0 output, the MLM mode with K was chosen for the association mapping of *ZmRCAβ* and *OsRCA* expression. Markers were identified as significantly associated with expression of *ZmRCAβ* or *OsRCA* by comparison with the Bonferroni threshold (*P* < 1/n, −log*P* > 5.75 for *ZmRCAβ*, −log*P* > 4.57 for *OsRCA*).

### Promoter-based association analysis

CTAB method was employed to extract genomic DNA (gDNA) of maize and rice leaves [48]. The promoters of *ZmRCAβ* and *OsRCA* were amplified using specific primers (Additional file 1: Table S1) and the PCR procedure as described in our previous study [20]. The sample PCR products were then purified and sequenced by BGI (Shanghai, China). Tested sequences were aligned using CLUSTALX version 1.83 [49]. The identification of DNA variations among these genotypes and the candidate-gene association analysis between polymorphisms and phenotypes were performed using Tassel 5.0 [46]. In the candidate-gene association analysis for the RCA gene promoter, all polymorphisms with a MAF ≥0.05 were considered [44].

### Promoter activity analysis in rice callus

Maize and rice RCA promoters, which are 1700 bp and 1500 bp in length, respectively, were amplified from genomic DNA with specific primers (Additional file 1: Table S1) added with the cutting sites of *Hin*dIII and *Sa1*I enzymes. The amplified fragment was separated on 1% agarose gels and purified with a gel extraction kit (Axygen) according to the manufacturer’s protocol. The purified fragment was fused with the GUS reporter gene in the binary vector pCAMBIA1381Z digested with *Hin*dIII and *Sa1*I to construct recombinant GUS vector. Promoter-less vector pCAMBIA1381Z and CaMV 35S promoter-driving GUS vector pCAMBIA1301 were used as negative and positive controls, respectively. The recombinant GUS vector, negative control, and positive control were individually transformed into rice callus via *Agrobacterium*-mediated transformation as described by Hiei et al. [50].

GUS activity was determined in the rice positive callus according to the method as described previously [51] with some modifications. Plant materials were immersed in 100 mM sodium phosphate buffer (pH 7.0) containing 1% Triton X-100, 1% DMSO, 10 mM EDTA and 0.5 mg/ml 5-bromo-4-chloro-3-indolyl glucuronide and incubated overnight at 37 °C. The staining solution was then removed, and the samples were dehydrated using 75% ethanol. GUS staining was observed under an Olympus SZX12 stereomicroscope and photographed with a digital camera (CoolSNAP, RS photometrics).

### Promoter activity analysis using dual luciferase assay

A dual luciferase assay vector pGreenII0800-LUC was used to analyze the activity of the target promoter. This vector contains firefly luciferase (LUC) reporter gene that can be driven by the target promoter and Renilla luciferase (REN) reporter gene driven by 35S. The purified DNA fragment of the target promoter was fused with LUC reporter gene in the vector digested with *Hin*dIII and *Sa1*I enzymes to construct the recombinant vector. The vector pGreenII0800-LUC without promoter insertion before LUC reporter gene was used as negative control. The recombinant and negative control vectors were individually transformed into maize and rice protoplasts or tobacco (*Nicotiana benthamiana*) leaves. In the protoplast transient expression experiments, the isolation of protoplasts from maize or rice green leaves, PEG-calcium transfection of plasmid DNA, and protoplast culture were performed according to standard protocols [52]. In the leaf transient expression experiment, tobacco plants were grown until at least six leaves were available. The transfection of plasmid DNA into tobacco leaves by infiltration with *Agrobacterium* and transient expression assay followed standard protocols [53].

The ratio of LUC and REN activity (LUC/REN) was used to reflect the activity of the target promoter. The LUC/REN value was determined using the dual luciferase reporter assay system (Promega). Briefly, the transformed protoplasts or homogenate prepared from transformed tobacco leaves were centrifuged at 12000×g for 15 s at room temperature, and the supernatant was removed. Next, 100 μl of passive lysis buffer was added for further homogenization. Twenty microliters of lysate was mixed with 100 μl of LAR II, and then the LUC activity was measured using a GloMax 20/20 luminometer (Promega). Finally, 100 μl of Top & Glo reagent was added to the reaction, and the REN activity was measured.

## Additional files

Additional file 1: **Table S1.** Primer pairs used in this study. (DOCX 108 kb)

Additional file 2: **Table S2.** Polymorphic sites in the promoter region of *ZmRCAβ* and *OsRCA*. (XLSX 9 kb)

Additional file 3: **Table S3.** Origin and haplotype of plant materials of maize and rice association populations in this study. (XLSX 11 kb)

Additional file 4: **Figure S1.** Activity of different types of RCA promoters. (A) Schematic diagrams of the GUS vector. GUS, β-glucuronidase; ter, terminator. (B) Histochemical staining of rice callus transformed with recombinant plasmids containing different types of *ZmRCAβ* and *OsRCA* promoters, positive control, and negative control*.* The result showed that GUS expression driven by the ZmHap1 promoter was stronger than that by ZmHap3 promoter, GUS expression driven by the OsHap1 promoter was stronger than that by the OsHap5 promoter, and the GUS expression driven by rice promoters was stronger than that by the maize promoters. (DOCX 14 kb)

Additional file 5: **Table S4.** SNP markers associated with the expression of *OsRCA* in association population. (XLSX 9 kb)

Additional file 6: **Figure S2.** GWAS for the expression of *OsRCA*. (A) Quantile-quantile plot for the GWAS under a mixed linear model (MLM). (B) Manhattan plot for the GWAS. The red line indicates a significant association signal (−log*P* > 4.57). (DOCX 248 kb)

Additional file 7: **Table S5.** Polymorphisms in the *OsRCA* promoter region significantly associated with gene expression. (XLSX 10 kb)

Additional file 8: **Figure S3**. *OsRCA* promoter haplotypes and the average *OsRCA* expression for each haplotype. (A) Haplotype analysis of the *OsRCA* promoter regions. The dark gray-shaded cells represent the favorable alleles. (B) The average expression of *OsRCA* for different haplotypes. Error bars represent the standard error. The number of data points used for calculating the standard error is the product of number of rice cultivars belonging to each haplotype and biological replicates of each cultivar. Multiple comparison of phenotypic data was performed using LSD test. Different English alphabet means significant difference at *P* < 0.05. (DOCX 878 kb)

Additional file 9: **Table S6.** SNP markers associated with the maize grain yield in the association population. (XLSX 36 kb)

Additional file 10: **Table S7**. *Cis*-acting elements in the RCA promoter region of C_3_ and C_4_ species. (XLSX 12 kb)

## Data Availability

All data generated or analysed during this study are included in this published article (and its supplementary information files).

## Abbreviations

ANOVA: analysis of variance
CCM: CO_2_-concentrating mechanism
eQTL: Expression quantitative trait locus
GUS: β-glucuronidase
GWAS: Genome-wide association analysis
*H*^*2*^: the broad-sense heritability
indel: insertion-deletion
LD: linkage disequilibrium
LUC: firefly luciferase
MAF: minor allele frequencies
MLM: the mixed linear model
*p*CO_2_: CO_2_ partial pressure
PEG: polyethylene glyco
RCA: Rubisco activase
REN: renilla luciferase
RIL: recombinant inbred line
Rubisco: Ribulose-1,5-bisphosphate carboxylase/oxygenase
SNP: single nucleotide polymorphisms
