# Supplementary material for: Genetic determinants controlling maize rubisco activase gene expression and a comparison with rice counterparts
Source: BMC Plant Biol. 2019 Aug 14;19:351. doi: 10.1186/s12870-019-1965-x (PMC6692957; doi:10.1186/s12870-019-1965-x)
Supplement: Supplementary file 1 — Table S1. Primer pairs used in this study. (DOCX 108 kb) [file 12870_2019_1965_MOESM1_ESM.docx]

**Table S1.** Primer pairs used in this study.

| Gene | Primers | For real-time RT-PCR | For promoter amplifying | For Vector construction^a^ |
| --- | --- | --- | --- | --- |
| *ZmRCAβ* | Forward | CCGAACTAAAAAGCACAAGAAATG | GTTGTCCACATCGCCAAGAA | ACGCGTCGACGTTGTCCACATCGCCAAGAA |
|  | Reverse | CAGCCATCGCCTTGAACCT | GCGCTAGCAGGTATGGTGGT | CCCAAGCTTGCGCTAGCAGGTATGGTGGT |
| *OsRCA* | Forward | CGTGACGGGCGTAT GGAGAAG | AAATATACAAGTTCAAG | ACAGTCGACAAATATACAAGTTCAAG |
|  | Reverse | GCACGAAGAGCGCCGAAGAA ATC | GTAGAGGATCACT | TCGAAGCTTGTAGAGGATCACT |
| *ZmActin* | Forward | GATGATGCGCCAAGAGCTG |  |  |
|  | Reverse | GCCTCATCACCTACGTAGGCAT |  |  |
| *OsActin* | Forward | CAACACCCCTGCTATGT ACG |  |  |
|  | Reverse | CATCACCAGAGTCCAACACAA |  |  |

^a^ The underlined hexamers GTCGAC and AAGCTT are the *Sal*I and *Hin*dIII restriction sites, respectively.
