# Supplementary material for: Genetic determinants controlling maize rubisco activase gene expression and a comparison with rice counterparts
Source: BMC Plant Biol. 2019 Aug 14;19:351. doi: 10.1186/s12870-019-1965-x (PMC6692957; doi:10.1186/s12870-019-1965-x)
Supplement: Supplementary file 4 — Figure S1. Activity of different types of RCA promoters. (A) Schematic diagrams of the GUS vector. GUS, β-glucuronidase; ter, terminator. (B) Histochemical staining of rice callus transformed with recombinant plasmids containing different types of ZmRCAβ and OsRCA promoters, positive control, and negative control. The result showed that GUS expression driven by the ZmHap1 promoter was stronger than that by ZmHap3 promoter, GUS expression driven by the OsHap1 promoter was stronger than that by the OsHap5 promoter, and the GUS expression driven by rice promoters was stronger than that by the maize promoters. (DOCX 14 kb) [file 12870_2019_1965_MOESM4_ESM.docx]

**
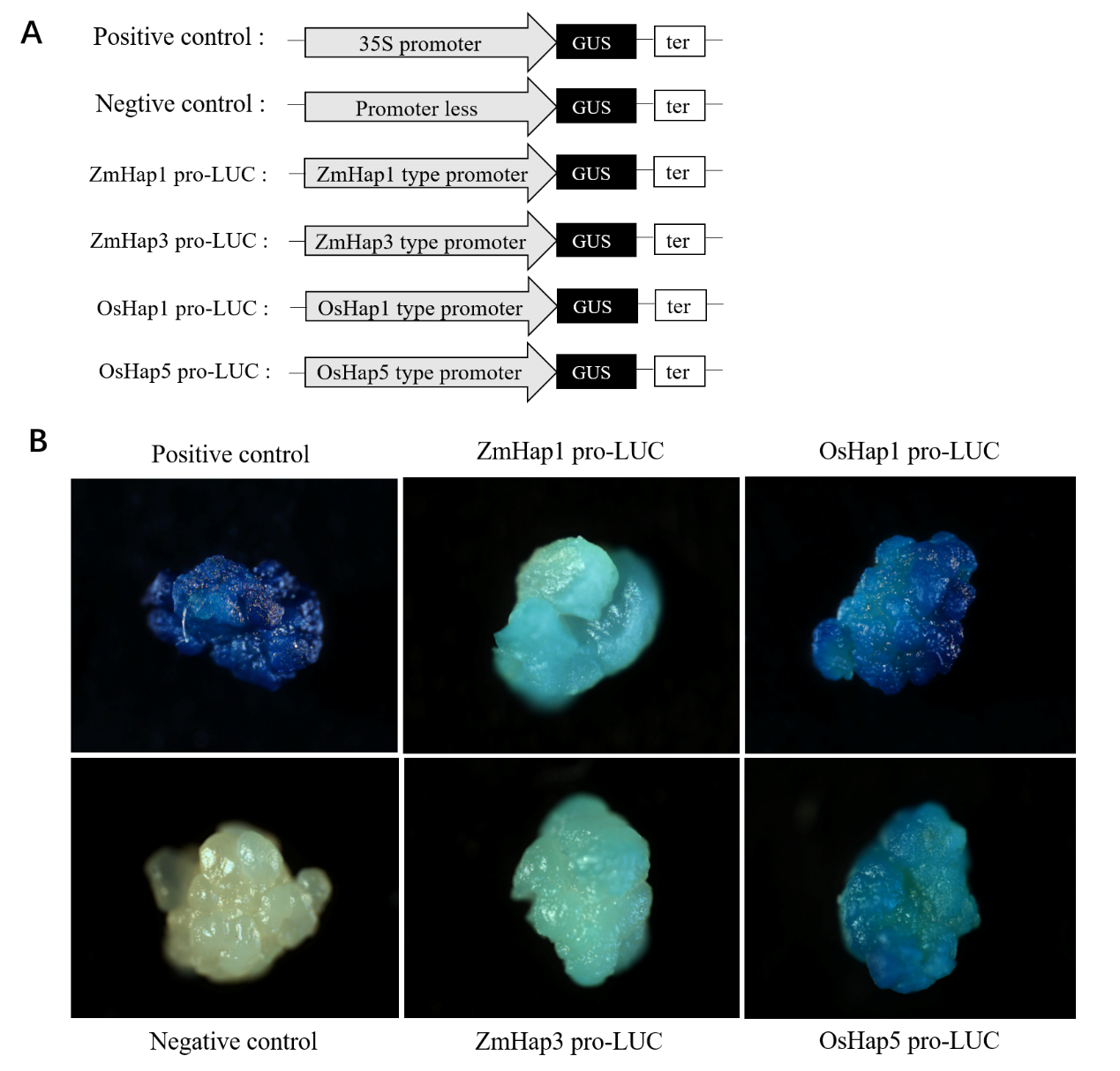
**

**Figure S1** Activity of different types of RCA promoters. (A) Schematic diagrams of the GUS vector. GUS, β-glucuronidase; ter, terminator. (B) Histochemical staining of rice callus transformed with recombinant plasmids containing different types of *ZmRCAβ* and *OsRCA* promoters, positive control, and negative control*.* The result showed that GUS expression driven by the ZmHap1 promoter was stronger than that by ZmHap3 promoter, GUS expression driven by the OsHap1 promoter was stronger than that by the OsHap5 promoter, and the GUS expression driven by rice promoters was stronger than that by the maize promoters.
