# Supplementary material for: Genetic determinants controlling maize rubisco activase gene expression and a comparison with rice counterparts
Source: BMC Plant Biol. 2019 Aug 14;19:351. doi: 10.1186/s12870-019-1965-x (PMC6692957; doi:10.1186/s12870-019-1965-x)
Supplement: Supplementary file 6 — Figure S2. GWAS for the expression of OsRCA. (A) Quantile-quantile plot for the GWAS under a mixed linear model (MLM). (B) Manhattan plot for the GWAS. The red line indicates a significant association signal (−logP > 4.57). (DOCX 248 kb) [file 12870_2019_1965_MOESM6_ESM.docx]

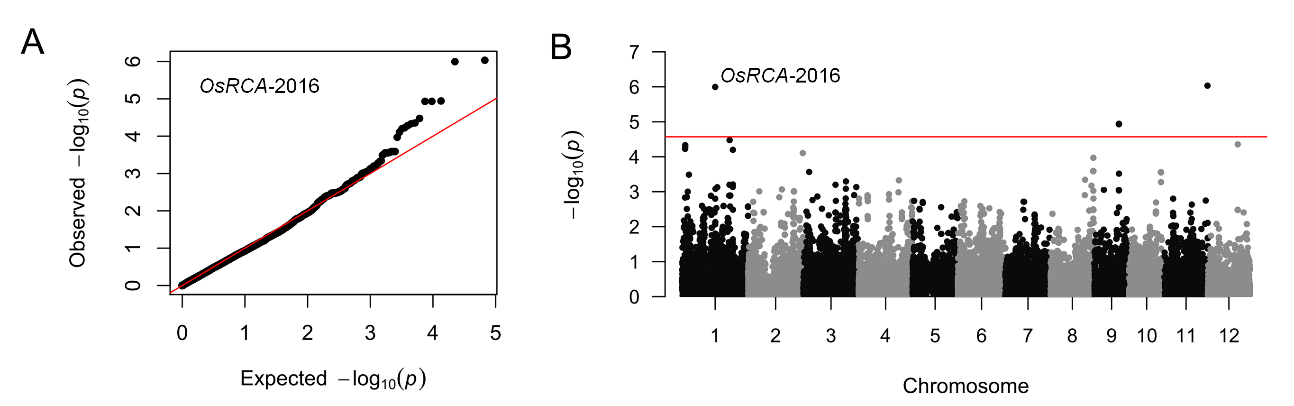


**Figure S2** GWAS for the expression of *OsRCA*. (A) Quantile-quantile plot for the GWAS under a mixed linear model (MLM). (B) Manhattan plot for the GWAS. The red line indicates a significant association signal (-log*P* > 4.57).
