# Supplementary material for: Genetic determinants controlling maize rubisco activase gene expression and a comparison with rice counterparts
Source: BMC Plant Biol. 2019 Aug 14;19:351. doi: 10.1186/s12870-019-1965-x (PMC6692957; doi:10.1186/s12870-019-1965-x)
Supplement: Supplementary file 8 — Figure S3. OsRCA promoter haplotypes and the average OsRCA expression for each haplotype. (A) Haplotype analysis of the OsRCA promoter regions. The dark gray-shaded cells represent the favorable alleles. (B) The average expression of OsRCA for different haplotypes. Error bars represent the standard error. The number of data points used for calculating the standard error is the product of number of rice cultivars belonging to each haplotype and biological replicates of each cultivar. Multiple comparison of phenotypic data was performed using LSD test. Different English alphabet means significant difference at P < 0.05. (DOCX 878 kb) [file 12870_2019_1965_MOESM8_ESM.docx]

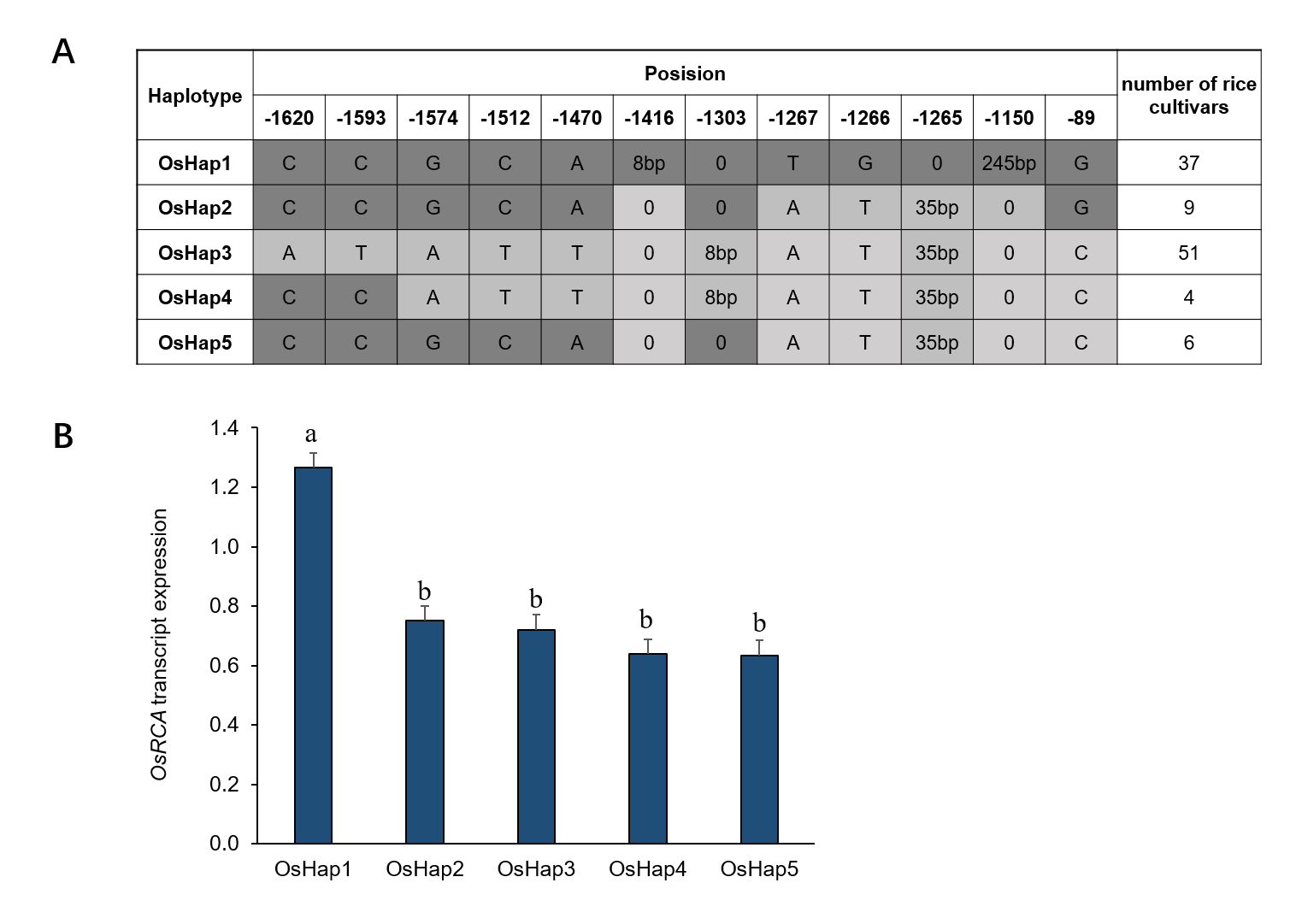


**Figure S3** *OsRCA* promoter haplotypes and the average *OsRCA* expression for each haplotype. (A) Haplotype analysis of the *OsRCA* promoter regions. The dark gray-shaded cells represent the favorable alleles. (B) The average expression of *OsRCA* for different haplotypes. Error bars represent the standard error. The number of data points used for calculating the standard error is the product of number of rice cultivars belonging to each haplotype and biological replicates of each cultivar. Multiple comparison of phenotypic data was performed using LSD test. Different English alphabet means significant difference at *P* < 0.05.
